# Age as a potential predictor of acute side effects during chemoradiotherapy in primary cervical cancer patients

**DOI:** 10.1186/s12885-022-09480-w

**Published:** 2022-04-07

**Authors:** Annica Holmqvist, Gabriel Lindahl, Rasmus Mikivier, Srinivas Uppungunduri

**Affiliations:** 1 Department of health, medicine and caring siences, Linköping University, Linköping, Sweden; 2 Department of Oncology, S-58185 Linköping, Sweden; 3 Regional Cancer Centre Southeast, Linköping, Sweden

**Keywords:** Age, Nausea/vomiting, Diarrhea, Weight loss, Chemoradiotherapy, Cervical cancer

## Abstract

**Background:**

Toxicity during chemoradiotherapy (CRT) in cervical cancer patients might limit the chances of receiving an optimal treatment and to be cured. Few studies have shown relationships between acute side effects and patient’s age. Here, the association between age and acute side effects such as nausea/vomiting, diarrhea and weight loss during CRT was analysed in cervical cancer patients.

**Methods:**

This study included 93 patients with primary cervical cancer stage IBI to IVA who received CRT from 2013 to 2019. The frequency of symptoms/toxicity grade was analysed by using the Common Terminology Criteria for Adverse Events (CTCAE) version 5.0.

**Results:**

Patients ≥ 52 years had a significantly higher frequency of nausea/vomiting and increased grade ≥ 3 toxicity during CRT compared to younger patients (*p* < 0.001, *p* = 0.001). Toxicity grade ≥ 3 of nausea/vomiting was associated with increased frequency of weight loss (*p* = 0.001), reduced ADL (*p* = 0.001) and dose modifications of both radiotherapy (RT) (*p* = 0.020) and chemotherapy (CT) (*p* = 0.030) compared to toxicity grade 2. The frequency of diarrhea (*p* = 0.015) and weight loss (*p* = 0.020) was higher in older patients compared to younger.

**Conclusions:**

Older patients have an increased risk of acute side effects as nausea/vomiting, diarrhea and weight loss. Age could be useful in predicting acute side effects in primary cervical cancer patients with CRT.

## Introduction

Cervical cancer is one of the leading causes of cancer related death in women, both, in Sweden and globally [1]. It often presents in advanced stages and despite aggressive treatment and recent advances in radiotherapy (RT), the risk of recurrence is still high [2]. Currently, primary chemo-radiotherapy (CRT) is the standard of care in patients with cervical cancer from stage IB2 to IVA [3, 4]. Further, postoperative CRT is recommended in cases of early-stage disease (IB1) with narrow surgical margin or lymph-node metastases [5, 6]. Acute toxic reactions caused by CRT are a common problem and often require dose modifications, which could potentially impair the curative effects of CRT. Previous studies have shown that treatment of cervix cancer patients with intensity modulation radiotherapy (IMRT) significantly reduced the level of gastrointestinal (GI) and hematological toxicity in the pelvic tissue in comparison to conventional RT [7–9].

Today, approximately half of the patients diagnosed with cervical cancer are younger than 50 years [3, 10, 11]. In recent studies, it has been shown that the frequency of acute toxic reaction was distributed equally between older and younger cancer patients [12–14]. A previous large meta-analysis of pelvic cancer patients with conventional RT showed that patients younger than 65 years had a higher frequency of nausea/vomiting compared to older patients [12]. Others found no significant difference in the frequency of nausea/vomiting between cervical cancer patients younger or older than 65 years [15]. A retrospective study involving primary cervical cancer patients treated with CRT showed that older patients had an increased risk of lymphatic and cardiologic toxicity. However, acute symptoms like nausea/vomiting during CRT, which are common clinical problems for these patients, were not included this study [13].

Diarrhea during CRT is a well know side effect and the frequency in cervical cancer patients varies between 68–96% [16–19]. A small number of studies have analysed the relationship between age and diarrhea during CRT in cervical cancer patients and found no significant association [12, 13, 15].

A weight loss of more than 5% during CRT in cancer patients is known to increase the risk of both malnutrition and morbidity [20]. Few studies have analysed the frequency of weight loss in primary cervical cancer patients during CRT [21, 22]. Further, the association between age and weight loss has not been studied before.

Adverse effects during CRT are a common problem that could affect the treatment and in consequence, affect the prognosis for these patients. Therefore, early interventions to reduce the acute toxicity may increase the compliance to CRT. The aim of this study was to investigate the frequency and grade of acute side effects as nausea/vomiting, diarrhea and weight loss in relation to age during CRT in primary cervical cancer patients.

## Methods

### Patients

This retrospective cohort study included 93 patients with primary cervical cancer stage IBI to IVA (Fédération Internationale de Gynécologie et d’Obstétrique (FIGO 2009)) who received CRT from October 2013 to April 2019 at the Department of Gynecologic Oncology, University Hospital, Linköping, Sweden. All patients with primary cervical cancer who received either curative or postoperative CRT were asked to participate in the study. All 93 patients had concurrent chemotherapy (CT). The treatment was given according to the national guidelines. Patients with dementia, who were unable to read and speak Swedish or not capable of receiving the standard CRT treatment due to poor performance status and/or co-morbidities were not included in the study. The regional ethical committee in Linköping, Sweden approved the study (Reference number: 2018/363–31, 2019/013–33). All patients had signed a written informed consent.

### Data

Data such as age, stage, grade, time of diagnosis, date of surgery, survival, type of CRT, compliance and side effects during CRT were obtained from the patients’ medical records (Table 1).

**Table 1:** Clinicopathological factors in relation to age in 93 cervical cancer patients with (CRT). The Chi-square (X^2^) test and the Fischer´s exact test was used for statistical analysis

Variables Age *n* = 93	< 52 years *n* = 47 (%)	≥ 52 years *n* = 46 (%)	*p*-value
**Stage**			
IB1-IB2	24 (51.1)	7 (15.2)	0.003
IIA1-IIB	16 (34.0)	27 (58.7)	
IIIA-IIIB	6 (12.8)	8 (17.4)	
IVA	1 (2.1)	4 (8.7)	
**Histology**			
Squamous cell carcinoma	33 (70.2)	37 (80.4)	0.413
Adenocarcinoma	12 (25.5)	7 (15.2)	
Adenoskvamous carcinoma	0	1 (2.2)	
Others	2 (4.3)	1 (2.2)	
**Hospitalisation**	10 (21.3)	25 (54.4)	0.001
Yes	37 (78.7)	21 (45.6)	
No			
Performance status (WHO)**			
0	43 (91.5)	36 (78.3)	0.068
1	3 (6.4)	10 (21.7)	
2	1 (2.1)	0	
Other diseases**			
No	45 (95.7)	29 (63.0)	< 0.001
Yes	2 (4.3)	17 (37.0)	
Diabetes	1 (2.1)	9 (19.6)	
Hypothyreosis	1 (2.1)	5 (10.9)	
Inflammatory bowel disease	0	1 (2.2)	
Cardiovascular disease	0	2 (4.3)	
**Type of surgery**			
No surgery	31 (66.0)	38 (82.6)	0.076
TAH + SOEB* + pelvic lymphadenectomy	14 (29.8)	5 (10.9)	
TAH + SOEB*	2 (4.2)	3 (6.5)	
**Type of (CRT)**			
2 Gy × 25 pelvis	10 (21.3)	8 (17.4)	0.635
1.8/2 Gy × 25 pelvis + cervical (BT) and/or boost	37 (78.7)	38 (82.6)	
**Distribution of (RT)**			
Extended-field RT	7 (14.9)	6 (13.0)	0.797
No extended-field RT	40 (85.1)	40 (87.0)	
Boost to pelvic/para-aortic lymph-nodes	16 (34.0)	15 (32.6)	0.883
No boost to pelvic/para-aortic lymph-nodes	31 (66.0)	31 (67.4)	
**Compliance (RT)**			
No interruption	46 (97.9)	38 (82.6)	0.014
Dose interruption/stopped treatment	1 (2.1)	8 (17.4)	
**Compliance (CT)**			
No interruption	32 (68.1)	20 (43.5)	0.017
Dose reduction/stopped treatment	15 (31.9)	26 (56.5)	

The primary target volume (PTV), (D98; minimum dose to 98% volume) and the RT doses to the rectum and bladder (D2cc; minimum dose in Gray (Gy) to 2% volume) were obtained retrospectively from the radiotherapy treatment planning system (Eclipse™ Treatment Planning Software v 16.00 from Varian Medical Systems). Of 93 patients, RT doses to the rectum and bladder were available for 87 patients. Data was missing for six patients due to incomplete registration at the time point for treatment. Blood tests, patient’s weight (kg) and an evaluation of toxicity were repeated weekly during CRT. The acute toxicity during the CRT was evaluated by stratifying for category of side effects and toxicity grade in accordance with the US National Cancer Institute, Common Terminology Criteria for Adverse Events (CTCAE) version 5.0 [23, 24]. The CTCAE toxicity for grade 3 of weight loss was set at ≥ 20%, which is, clinically, a very high level of weight loss for these patients. In our study, no patient had such a high level of weight loss (max 11.9%) and therefore only grade 1 and 2 toxicity are presented here. Each patients’ medical record was studied retrospectively from the start of the CRT to the end of the treatment. The highest grade of toxicity during the CRT treatment according to CTCAE was documented and used for further analyses. The frequency/toxicity of side effects were further studied in relation to the age subgroups < 52 years and ≥ 52 years. These two subgroups were chosen since the median age of the patients in study was 52 years, which resulted in two sub-groups with equal number of patients. Similar categorization have been used by others [10, 11].

### Statistics

The Chi-square (X^2^) test and the Fischer´s exact test was used to analyse clinical and pathological factors and to study the differences between the frequency and toxicity grade of the side effects in relation to age. A t-test (independent by groups) were used to calculate the differences in RT doses for PTV and the RT doses to the rectum and bladder related to age. The differences in the overall survival (OS) and progression free survival (PFS) between the two age groups in relation to the side effects were analysed by using the univariate Cox proportional hazard model. Survival curves were calculated according to the Kaplan–Meier method. The software program STATISTICA (version 13.5) was used for the Statistical analyses. The tests were two-sided and a *p*-value of *p* < 0.05 was considered statistically significant.

## Results

### Study population

Ninety-three patients with primary cervical cancer with CRT were included in the study. Most of the patients (79.6%) were in stage IB1 to IIB at the time of diagnosis. Squamous cell carcinoma was present in 75.3% of the patients and adenocarcinoma in 20.4% (Table 1). The median age of the patients was 52 years (range 28–79). The mean follow-up for the surviving patients was 46.2 months (range 9–85 months). At the latest follow, 24 (25.8%) out of 93 patients had local and/or distant recurrence and 19 (20.4%) had deceased.

Further, the OS and PFS were compared between patients older and younger than 52 years, but no significant association was found for either OS or PFS between the two subgroups of patients aged ≥  < 52 years (*p* = 0.878, *p* = 0.432).

### Treatment

The external beam radiotherapy was given with 45–50 Gy in 25 fractions to the pelvis in combination with CT. Of the 93 patients, 19.4% received pelvic RT alone and 80.6% pelvic RT + cervical brachytherapy (BT) and/or boost. All treatments were delivered with either IMRT or volumetric modulated arc therapy (VMAT). The concomitant CT consisted of Cisplatin 40 mg/m^2^ given once a week. The mean number of CT treatments was 4.5 (range 2–6). Regarding the RT treatment, eight patients (8.6%) had dose interruption with prolongation of the time for RT treatment. One patient (1.1%) stopped due to side effects (Table 1).

There were no differences in the mean RT doses for PTV D98% or the mean D2cc (Gy) to the rectum and bladder between patients < 52 and ≥ 52 years. The mean PTV D98% for the younger patients compared to the older patients was 85.48 (± SD 9.74) Vs.84.24 (± SD 12.60)*,* (*p* = 0.610). The mean D2cc (Gy) to the rectum and bladder for patients < 52 years compared to patients ≥ 52 years were 64.0 Gy (± SD 11.7) Vs. 65.1 Gy (± SD 11.3), (*p* = 0.667) and 67.1 Gy (± SD 14.4) Vs. 70.62 Gy (± SD 17.6), (*p* = 0.314).

### Frequency/toxicity of acute side effects in all patients during chemoradiotherapy

Of the 93 patients with primary cervical cancer a total of 76 patients had acute side effects of nausea/vomiting (81.7%, Table 2), where 27 (35.5%) of these patients with symptoms had grade ≥ 3 toxicity (Table 3). Patients with grade ≥ 3 toxicity of nausea/vomiting had a significantly increased frequency of weight loss (*p* = 0.001), reduced ADL (*p* = 0.001) and dose modifications for both RT/CT (*p* = 0.020, *p* = 0.030) compared to patients with grade 2 toxicity (Table 4).

**Table 2:** Number of patients with side effects in relation to age during chemoradiotherapy (CRT) in 93 cervical cancer patients

Acute side effects	Agen = 93 (%)	< 52 years *n* = 47 (%)	≥ 52 years *n* = 46 (%)	*p*-value
**Nausea/vomiting**				
Yes	76 (81.7)	32 (68.1)	44 (95.7)	< 0.001
No	17 (18.3)	15 (31.9)	2 (4.3)	
**Diarreha**				
Yes	81 (87.1)	37 (78.7)	44 (95.7)	0.015
No	12 (12.9)	10 (21.3)	2 (4.3)	
**Weight loss**				
Yes ≥ 5%	28 (30.1)	9 (19.1)	19 (41.3)	0.020
No < 5%	65 (69.9)	38 (80.9)	27 (58.7)	
**Limiting ADL**				
Yes	20 (21.5)	3 (6.4)	17 (37.0)	< 0.001
No	73 (78.5)	44 (93.6)	29 (63.0)	
**Anemia**				
Yes	70 (75.3)	35 (74.5)	35 (76.1)	0.856
No	23 (24.7)	12 (25.5)	11 (23.9)	
**Trombocytopenia**				
Yes	67 (62.0)	28 (59.6)	39 (84.8)	0.007
No	26 (28.0)	19 (40.4)	7 (15.2)	
**Leucopenia**				
Yes	47 (50.5)	22 (46.8)	25 (54.3)	0.467
No	46 (49.5)	25 (53.2)	21 (45.7)	
**Febrile neutropenia**				
Yes	16 (17.2)	5 (10.6)	11 (23.9)	0.090
No	77 (82.8)	42 (89.4)	35 (76.1)	
**Pain**				
Yes	10 (10.8)	6 (12.8)	4 (8.7)	0.526
No	83 (89.2)	41 (87.2)	42 (91.3)	
**Urinary symptoms**				
Yes	21 (22.6)	7 (14.9)	14 (30.4)	0.073
No	72 (77.4)	40 (85.1)	32 (69.6)	

The Chi-square (X^2^) test and the Fischer´s exact test was used to analyse the frequency of patients with acute side effects in the age subgroups ≥  < 52 years

**Table 3:** The number of patients experiencing various acute toxicities during chemoradiotherapy (CRT) treatment according to CTCAE (version 5.0) in 93 primary cervical cancer patients

Acute toxicity grade	< 52 years *n* = 47 (%)	≥ 52 years *n* = 46 (%)	*p*-value
**Nausea/vomiting**			
Grade 2	15 (31.9)	14 (30.4)	0.001
Grade ≥ 3	3 (6.4)	24 (52.2)	
**Diarreha**			
Grade 2	24 (51.1)	25 (54.3)	0.009
Grade ≥ 3	3 (6.4)	17 (37.0)	
**Weight loss**			
Grade 1	10 (21.3)	17 (37.0)	0.421
Grade ≥ 2	0	2 (4.3)	
**Limiting ADL**			
Grade 2	2 (4.3)	1 (2.2)	0.046
Grade ≥ 3	1 (2.1)	16 (34.8)	
**Anemia**			
Grade 2	16 (34.0)	12 (26.1)	0.624
Grade ≥ 3	2 (4.3)	1 (2.2)	
**Trombocytopenia**			
Grade 2	9 (19.1)	8 (17.4)	0.247
Grade ≥ 3	3 (6.4)	7 (15.2)	
**Leucopenia**			
Grade 2	12 (25.5)	9 (19.6)	0.232
Grade ≥ 3	9 (19.1)	14 (30.4)	
**Febrile neutropenia**			
Grade 2	0	0	1.000
Grade ≥ 3	5 (10.6)	11 (23.9)	
**Pain**			
Grade 2	1 (2.1)	2 (4.3)	0.333
Grade ≥ 3	5 (10.6)	2 (4.3)	
**Urinary symptoms**			
Grade 2	4 (8.5)	9 (19.6)	0.571
Grade ≥ 3	1 (2.1)	1 (2.2)	

The Chi-square (X^2^) test and the Fischer´s exact test was used to analyse the grade of toxicity between the age subgroups ≥  < 52 years regarding several different type of acute side effects

**Table 4:** Acute toxicity of nausea/vomiting in relation to the side effects weight loss, ADL, compliance to RT/CT in primary cervical cancer patients

Acute side effects	Nausea/vomiting *n* = 56 (%)	CTCAE 2 *n* = 29 (%)	CTCAE ≥ 3 *n* = 27 (%)	*p*-value
**Weight loss**				
Yes ≥ 5%	27 (48.2)	8 (27.6)	19 (70.4)	0.001
No < 5%	29 (51.8)	21 (72.4)	8 (29.6)	
**Limiting ADL**				
Yes	17 (30.4)	3 (10.3)	14 (51.9)	< 0.001
No	39 (69.6)	26 (89.7)	13 (48.1)	
**Compliance RT**				
Dose modification/stopped treatment	8 (14.3)	1 (3.4)	7 (25.9)	0.020
No interruption	48 (85.7)	28 (96.6)	20 (74.1)	
**Compliance CT**				
Dose modification/stopped treatment	31 (55.4)	12 (41.4)	19 (70.4)	0.030
No interuption	25 (44.6)	17 (58.6)	8 (29.6)	

A Chi-square (X^2^) test and a Fischer´s exact test was used for the statistical analyses

Eighty-one (87.1%) of the 93 patients had diarrhea during the CRT (Table 2) out of which 20 patients (24.7%) had grade ≥ 3 toxicity (Table 3). Twenty-eight (30.1%) of 93 patients, had ≥ 5% weight loss (Table 2).

### Frequency/toxicity of acute side effects in relation to age during chemoradiotherapy

**Fig. 1:**
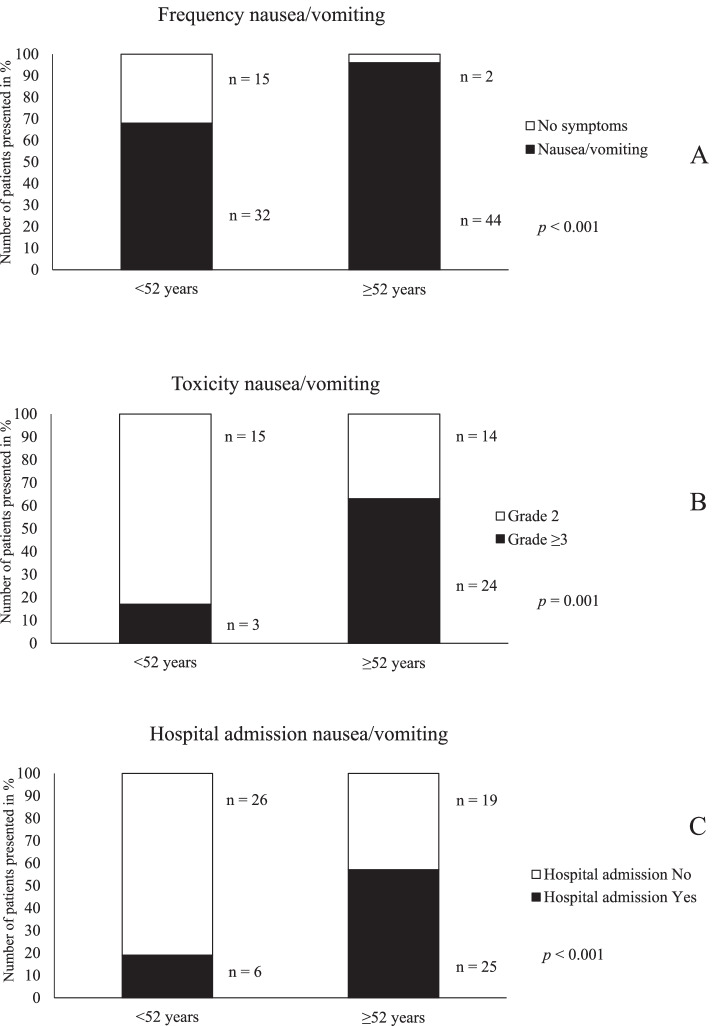
Patients age in relation to frequency of side effects as nausea/vomiting (**A**), grade of toxicity (**B**) and number of patients with nausea/vomiting who needed hospital admission (**C**) in primary cervical cancer patients with CRT

**Fig. 2:**
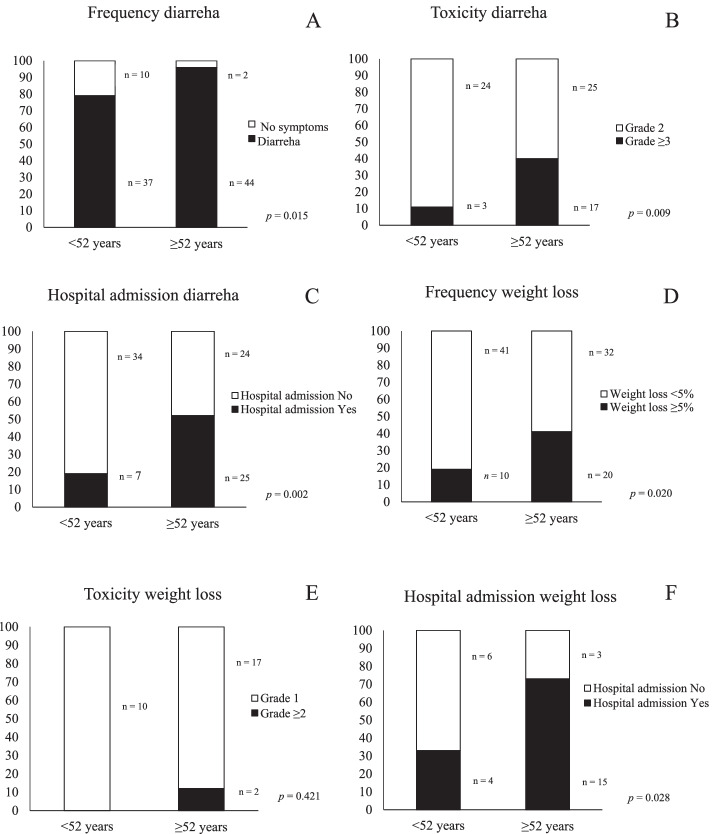
Patient’s age in relation to frequency (**A**) and toxicity grade of diarrhea (**B**) and number of patients who needed acute hospital admission due to diarrhea (**C**). Patient’s age in relation to frequency (**D**) and toxicity grade of weight loss (**E**) and number of patients who needed hospital admission due to weight loss (**F**) in primary cervical cancer patients with CRT. All figures are presented as number of patients in percent

The frequency of acute side effects and grade of toxicity during CRT was further studied in relation to age in cervical cancer patients. Patient’s ≥ 52 years had a significantly higher frequency of nausea/vomiting during CRT compared to younger patients (*p* < 0.001, Table 2, Fig. 1A). In addition, there was an age-related increase in the grade ≥ 3 toxicity from 6.4% in patients < 52 to 52.2% in patients ≥ 52 years (*p* = 0.001, Table 3, Fig. 1B). More than half of the 44 patients ≥ 52 years (56.8%, *n* = 25) needed acute hospital admission due to side effects as nausea/vomiting during the CRT compared to 18.8% (*n* = 6) of the 32 patients < 52 years (*p* = 0.001, Fig. 1C). A higher frequency and toxicity grade of diarrhea was seen in older patients compared to younger (*p* = 0.015, *p* = 0.009, Tables 2 and 3, Fig. 2A-B). In addition, older patients with diarrhea were admitted for acute hospital care more often than younger patients (*p* = 0.002, Fig. 2C). Patients ≥ 52 years had a significantly higher frequency of weight loss ≥ 5% compared to patients < 52 years (*p* = 0.020, Table 2, Fig. 2D). No association was seen between age and toxicity grade of weight loss, probably due to low number of cases (*p* = 0.421, Table 3, Fig. 2E). Finally, older patients with weight loss ≥ 5% had an increased risk of acute hospital admission compared to patients < 52 years (*p* = 0.028, Fig. 2F).

Further, the acute side effects as nausea/vomiting, diarreha and weight loss were studied in relation to tumour stage and co-morbidity in the age sub-groups <  ≥ 52 years, separately, but no significant differences were found (*p* > 0.05).

### Frequency/toxicity of acute side effects in relation to radiotherapy

Next, the relationship between RT treatment and side effects were analysed in the cervical cancer patients. Patients with pelvic RT + cervical BT and/or boost tended to have a higher frequency of grade ≥ 3 toxicity of nausea/vomiting compared to patients with pelvic RT alone (53.2% vs 22.2%, *p* = 0.089). Neither was there any significant relation between nausea/vomiting, diarrhea or weight loss and extended-field RT or pelvic/para-aortic boost to lymph-nodes (*p* > 0.05).

## Discussion

Adverse effects during CRT are common occurrences in cervical cancer patients and often lead to disruptions of the treatment, which can potentially have a negative effect on the prognosis. Therefore, early detection and mitigation of the adverse effects caused by CRT is important. Previous studies do not provide a clear picture of the relationship between CRT, age and side effects [12–15]. The aim of this study was to analyse common acute side effects such as nausea/vomiting, weight loss and diarrhea in relation to age during CRT in primary cervical cancer patients.

In the present study, we showed that a majority of the patients (81.7%) had symptoms of nausea/vomiting during CRT. We also showed that older patients had a significantly higher frequency of nausea/vomiting during CRT compared to younger patients. Few studies have analysed the relationship between acute side effects as nausea/vomiting, age and pelvic RT/CRT and conflicting results have been presented. Chakraborty et al. (2014) studied primary cervical cancer patients with CRT and found no significant difference between patients younger and older than 65 years with regards to nausea/vomiting [15]. Pignon et al. (1997) showed a reverse relationship compared to ours, with younger patient showing a higher frequency of nausea/vomiting compared to older patients. However, several differences between the two studies including stratification into many age groups, heterogeneous inclusion of many pelvic cancer types and differences in RT treatment could account for these discrepancies [12]. Finally, a meta-analysis on cervical cancer patients with CRT did not include variables as nausea/vomiting at all, which is rather surprising since nausea/vomiting are clinically very common problems for these patients during CRT [13].

Further, we also showed that the toxicity grade of nausea/vomiting during CRT was significantly related to age. In the older patients, grade ≥ 3 toxicity was present in 52.2% of the patients compared to 6.4% in the younger patients. This is a much higher frequency of grade ≥ 3 toxicity in the older patients compared to Chakraborthy et al. (2014) where the grade ≥ 3 toxicity was present in 14.0% of the younger and in 8.7% of the patients older than 65 years [15]. Several differences between the study by Chakraborty et al. (2014) and the current study could explain these discrepancies. In our study, there were a lower number of patients who interrupted the RT treatment (0% vs 21.7%) and all patients received concurrent CT (100% vs. 65.2%). More patients in our study had extended-field RT (13.5% vs 4.3%) and there were also differences in the cut-off value for the age subgroups compared to the other study [15].

We also showed that more than half of the patients in the older group 56.8% needed hospital admission due to nausea/vomiting during CRT compared to only 18.8% in the younger group. Waddle et al. (2015) studied 1116 patients with several types of cancers who received EBRT in 2010 and showed that 20% needed unplanned hospital admission due to acute side effects caused by the treatment [25], which is in line with the frequency of younger patients admitted for acute hospital care in our study. However, in our study, the frequency of hospital admission for patients in the older group was much higher. The patients with toxicity grade ≥ 3 of vomiting/nausea had a significantly increased frequency of weight loss, reduced ADL and dose modifications of both RT/CT during CRT compared to patients with grade two toxicity. Thus, we can conclude that, patients ≥ 52 years have an increased risk of side effects as nausea/vomiting, which leads to a higher number of hospital admissions, reduced ADL, increased risk of weight loss and a higher amount of dose modifications of both RT and CT. Therefore, a regular follow up during CRT and early interventions are needed to mitigate these side effects.

Diarrhea during CRT is a well know side effect and the frequency in cervical cancer patients varies from 68.0% to 96.2% [16–19]. In line with previous reports, we showed that 87.1% of our 93 patients had symptoms of diarrhea. In recent studies, no significant relationships have been found between frequency/toxicity of diarrhea and age [12, 13, 15]. Here, we showed that, both, the frequency and toxicity of diarrhea was significantly increased in older patients compared to younger. Further, the incidence of diarrhea lead to more acute hospital admissions in older patients compared to younger.

A weight loss of more than 5% in cancer patients during CRT is associated with an increased risk of malnutrition and mortality [20]. Few have analyzed the symptoms of weight loss in cervical cancer patients during CRT [21] and no previous report has studied the association between weight loss and age in primary cervical cancer patients with CRT. Here, we showed that the frequency of weight loss was significantly increased in the older patients. Further, a weight loss of more than 5% leads to a higher risk of acute hospital admission for older patients. We would like to propose that age could be used to predict side effects as diarreha and weight loss in cervical cancer patients with CRT.

Treatment prolongation and unplanned interruption of CRT have a negative impact on OS in cancer patients [26, 27]. One major cause for this is the development of acute side effects during treatment. In our study, we had a higher frequency and more severe grade of side effects, especially nausea/vomiting in the older subgroup of patients, compared to previous studies [13, 15]. However, despite our findings we could not see any differences in survival between the younger and older patient subgroups. The reduced frequency of treatment interruptions/prolongations could be due to careful monitoring and supportive hospital admissions/medications. This underlines the importance of not only preventing severe side effects but also having a sound supportive strategy, including hospital admission to manage these side effects. As a next step, we plan to initiate an intervention program to study if we can reduce the acute side effects for patients at risk.

Next, different types of pelvic RT treatments were analysed in relation to nausea/vomiting, diarrhea and weight loss. Here we found a trend towards increased toxicity grade ≥ 3 of nausea/vomiting in patients with pelvic RT + cervical BT and/or boost compared to patients with pelvic RT alone. As far as we know no previous study has analysed the relationship between the acute side effect nausea/vomiting and type of pelvic RT. In one previous study 57 patients received pelvic RT + cervical BT and nine patients had only pelvic RT. Although the frequency of nausea/vomiting was presented in this study, no comparison was made between patients who received pelvic RT + cervical BT and pelvic RT alone [15]. No significant relationships were seen between weight loss, diarreah and type of pelvic RT in our study. We propose that pelvic RT + cervical BT and/or boost might contribute to the development of the side effect, nausea/vomiting due to a high RT dose affecting the small intestine located close to the cervix. Future studies, will focus on studying the RT doses in relation to the organs at risk in the pelvic area, especially concerning the treatment dose directed towards the small intestine (bowel bag).

It has been shown that older women are more often diagnosed with an advanced tumor stage compared to younger women [28]. In line with previous reports, the elderly patients in our study had a higher frequency of advanced tumors compared to the younger patients. A more advanced tumour stage might require treatment with higher doses of RT, with a corresponding increase in side effects as shown by Moore et al. (2016) in a meta-analysis of 1319 cervical cancer patients [13]. However, in this study, there were no significant differences in the mean PTV doses given to the pelvis or between the RT doses administered to the rectum and bladder between the age subgroups < or ≥ 52 years. In this material, in contrast to others, few patients [15] had co-morbidities that could affect the frequency/toxicity of the side effects initiated by the CRT treatment. Also, no significant association was found between co-morbidities and the side effects like nausea/vomiting, diarreha and weight loss when the age sub-groups were analysed separately.

These results make us suggest that the observed differences in frequency/toxicity of the side effects between the two age subgroups is not related to the RT doses. Furthermore, tumor stage does seem to affect risk of having side effects as nausea/vomiting, diarreah and weight loss in subgroup analyses of patients < or ≥ 52 years.

Our study was retrospective and based on a relatively small sample size, which can be a limitation. The study was not randomized and the data was collected from a single cancer care unit. However, the patients came from a relatively large catchment area (~ 1.5 million inhabitants) and our conclusions are based on real world data, reflecting the normal clinical spectra of patient with different ages and co-morbidities.

In conclusion, patient’s ≥ 52 years have an increased risk of side effects as nausea/vomiting, diarrhea and weight loss. Toxicity related to nausea/vomiting was associated with increased risk of weight loss, reduced ADL and dose modifications of CT/RT. We suggest that age can be used to predict acute side effects in primary cervical cancer patients with CRT. These results might help us in the future to identify the patients who need early interventions.

## Data Availability

The datasets used and/or analyzed during the current study available from the corresponding author on reasonable request.
